# The vestibular contribution to the head direction signal and navigation

**DOI:** 10.3389/fnint.2014.00032

**Published:** 2014-04-22

**Authors:** Ryan M. Yoder, Jeffrey S. Taube

**Affiliations:** ^1^Department of Psychology, Indiana University – Purdue University Fort WayneFort Wayne, IN, USA; ^2^Department of Psychological and Brain Sciences, Center for Cognitive Neuroscience, Dartmouth CollegeHanover, NH, USA

**Keywords:** vestibular, semicircular canals, otolith organs, spatial orientation, navigation

## Abstract

Spatial learning and navigation depend on neural representations of location and direction within the environment. These representations, encoded by place cells and head direction (HD) cells, respectively, are dominantly controlled by visual cues, but require input from the vestibular system. Vestibular signals play an important role in forming spatial representations in both visual and non-visual environments, but the details of this vestibular contribution are not fully understood. Here, we review the role of the vestibular system in generating various spatial signals in rodents, focusing primarily on HD cells. We also examine the vestibular system’s role in navigation and the possible pathways by which vestibular information is conveyed to higher navigation centers.

The survival of most species depends on the ability to navigate accurately within the environment. Accurate navigation depends on neural representations for location and direction, as well as distance traveled. These spatial signals are encoded by place cells, grid cells, border cells, and head direction (HD) cells (O’Keefe and Nadel, 1978; Taube, 2007; Moser et al., 2008; Lever et al., 2009). It is well known that external (allothetic) visual cues dominantly control these representations, but self-movement (idiothetic) cues can gain control when familiar visual cues are unavailable (O’Keefe and Conway, 1978; Hill and Best, 1981; Muller and Kubie, 1987; O’Keefe and Speakman, 1987; Quirk et al., 1990; Goodridge and Taube, 1995; Taube and Burton, 1995; Yoder et al., 2011a). These idiothetic cues include neural representations of the rate of linear movement through the environment, as well as the rate and direction (clockwise vs. counter-clockwise) the head is turning (McNaughton et al., 1989; Bassett and Taube, 2001; Sharp et al., 2001; Jacobs et al., 2010). Some of this information appears to originate in the vestibular system, and recent studies provide insight into the role of vestibular signals in spatial functions. This review therefore focuses on the contribution of vestibular information to navigation and the allocentric spatial signals in animals, with a primary emphasis on the HD signal in rodents.

## VESTIBULAR INVOLVEMENT IN NAVIGATION: BEHAVIORAL STUDIES

The vestibular system includes the semicircular canals and otolith organs, which sense angular and linear acceleration, respectively. At first glance, this self-movement information would not appear to be necessary for navigation under light conditions, given the predominant reliance on visual cues. However, humans with bilateral vestibular dysfunction are impaired at virtual navigation tasks, suggesting vestibular signals are necessary for navigation even in purely visual environments (Brandt et al., 2005). A more recent study found that humans were able to accurately use self-motion cues to accurately update their perceived orientation during active walking or passive transport, but were impaired when they walked in place (Frissen et al., 2011). This finding suggests that the vestibular detection of head movement is the critical factor in the perception of orientation in this task, given that motor efference signals would have been present during walking in place. The reader should consult Israël and Warren (2005) for a review of studies using human subjects that examines the role of the vestibular system in spatial orientation. A vestibular contribution to spatial signaling has also been found in non-human primates. For example, inertial motion influenced the activity of approximately two-thirds of the dorsal medial superior temporal area (MSTd) neurons that responded to optic flow cues, but this influence was absent in animals with bilateral vestibular lesions (Gu et al., 2007). Additionally, numerous rodent studies over the past few decades (**Table 1**) indicate that vestibular signals do influence landmark navigation, suggesting that visual information alone is not sufficient for accurate spatial performance. One possibility is that vestibular lesions disrupt behavior by eliminating the vestibulo-ocular reflex (VOR), which could affect visual acuity during active locomotion. However, if this were the case, we would expect deficits on tasks that require navigation relative to distal visual cues. This deficit does occur for some tasks, but intact landmark navigation has been reported in vestibular lesioned animals (e.g., Stackman and Herbert, 2002; Wallace et al., 2002). It is therefore unlikely that the behavioral deficits described below are attributed to the loss of the VOR.

**Table 1:** Effects of vestibular system manipulations on navigation and spatial signals in rodents.

Type	Manipulation	Result	Reference
Behavior	Vestibular nerve cauterization	Impaired T-maze alternation	Potegal et al. (1977)
	Vestibular nuclei lesion	Impaired return from passive transport	Miller et al. (1983)
	Passive rotation	Impaired working and reference memory (water maze)	Semenov and Bures (1989)
	Sodium arsanilate	Impaired working memory (radial maze)	Ossenkopp and Hargreaves (1993)
	Labyrinthectomy	Impaired reference memory (radial maze)	Russell et al. (2003a)
	Sodium arsanilate	Impaired navigation w/o visual cues (square arena)	Stackman and Herbert (2002)
	Sodium arsanilate	Impaired food-hoarding (homing) in darkness	Wallace et al. (2002)
	Vestibular aspiration	Impaired food-hoarding (homing) in darkness	Zheng et al. (2009)
	Slow rotation	Impaired pup retrieval (homing) in darkness	Mittelstaedt and Mittelstaedt (1980)
	Vestibular ablation + passive rotation	Impaired spatial discrimination (radial maze)	Matthews et al. (1989)
	Sodium arsanilate	Impaired working and reference memory (radial maze); impaired Y-maze alternation	Besnard et al. (2012)
	Utricle dysfunction	Impaired movement in darkness	Avni et al. (2009)
	Otolith dysfunction	Impaired working and reference memory (radial maze)	Kirby and Yoder (2012)
	Otolith dysfunction	Impaired place recognition (Y-maze)	Machado et al. (2012)
Electrophysiology	Labyrinthectomy	Attenuated hippocampal theta rhythm	Russell et al. (2006)
	Passive rotation	Induced theta rhythm	Shin (2010)
	Passive rotation	Induced theta rhythm	Tai et al. (2012)
	Sodium arsanilate	Eliminated HD signal	Stackman and Taube (1997)
	Tetrodotoxin	Eliminated HD and place cell signals	Stackman et al. (2002)
	Labyrinthectomy	Eliminated place cell signal	Russell et al. (2003b)
	Inverted orientation in microgravity	Eliminated HD signal	Taube et al. (2004)
	Microgravity (space flight)	No effect on place cell activity	Knierim et al. (2000)
	Inverted navigation	Disrupted HD signal	Calton and Taube (2005)
	Otolith dysfunction	Degraded HD signal	Yoder and Taube (2009)
	Passive rotation	Post-rotational activation of HD cell	Taube (2004) Barany Society Meeting (Figure 2)
	Plugged semicircular canal	Eliminated directional tuning of HD cells	Muir et al. (2009)
	Absence of horizontal canals	Absence of HD cells	Taube and Valerio (2012)

Vestibular involvement in rodent navigation is indicated by spatial deficits following vestibular system lesions or stimulation. One study investigated spontaneous alternation in rats that received bilateral cauterization of the vestibular nerve (Potegal et al., 1977). Spontaneous alternation is the behavior displayed by animals performing a repetitive T-maze task where both left and right arms are always baited for each trial. The animals normally alternate their choices on each trial between the two arms (left vs. right), and impairments are indicated by repeated entries to the same arm. This study used Douglas’s (1966) two-maze procedure, where two identical T-mazes were positioned parallel to one another in different rooms. Vestibular lesioned animals showed profound impairments relative to control groups, suggesting an important vestibular contribution on the performance of this spatial task. Another study confirmed the vestibular system’s role in navigation performance with a “return from passive transport” task that involved transporting water-deprived rats to points at increasing distances from a water spout, and then evaluating their ability to return directly to the start point (Miller et al., 1983). Blinded rats and those that received cerebellar cortex lesions were able to return successfully from greater distances than rats with lesions of the vestibular nuclei, although the vestibular lesioned animals performed better than predicted by chance.

Activation or lesion of the vestibular system also appears to disrupt spatial learning on tests of spatial working (within-trials) and reference (across-trials) memory. A period of rotation immediately before retrieval trials or after acquisition trials failed to disrupt the ability to find the platform location on working and reference memory versions of a water maze task, although post-rotatory effects increased escape latency (Semenov and Bures, 1989). However, when animals were rotated for 30 s while they were on the platform, the rotation disrupted later retrieval only when distal visual cues were available, suggesting vestibular activation can disrupt the use of spatial cues for navigation. Another study showed that transtympanic injections of sodium arsanilate, which destroys vestibular hair cells, impaired spatial working memory on an eight-arm radial maze, as indicated by greater latency and more revisits to arms (Ossenkopp and Hargreaves, 1993). The vestibular system is also important for spatial reference memory on the radial arm maze, as rats with bilateral vestibular lesions were impaired at finding a single baited arm (Russell et al., 2003a). A recent study confirmed the vestibular involvement in spatial learning, showing increased working and reference memory errors on the radial maze and reduced spontaneous alternation on a Y-maze following bilateral sodium arsanilate injections to the vestibular labyrinth (Besnard et al., 2012). Together, these results suggest that, despite the predominant reliance on visual cues, the vestibular system provides a necessary signal that complements visual information during landmark navigation.

In the absence of familiar landmarks, navigation is guided by self-movement cues including motor efference copy, optic flow, proprioception, or vestibular signals. Several tasks have been used to test the importance of the vestibular contribution to this type of navigation. The food-hoarding task requires animals to exit a start box and explore a circular table in search of food and, upon finding the food, carry it directly back to the start box where the food is consumed. This “homing” task is often conducted in both light and darkness; the ability to return directly to the start box in light is thought to involve landmark navigation whereas the direct return in darkness is thought to require an online record of self-movement cues throughout the outbound journey. Vestibular involvement in this form of navigation is indicated by impaired homing in darkness in rats with bilateral sodium arsanilate injections into the vestibular labyrinth (Wallace et al., 2002). Vestibular lesioned and control rats performed similarly in light, whether the start location was cued or hidden. However, vestibular lesioned rats were markedly impaired in darkness, suggesting the vestibular signals are critical for non-visual navigation. A later study confirmed these findings, and further demonstrated that the effects of vestibular dysfunction persist for at least 5 months after the lesion (Zheng et al., 2009). Importantly, the initial heading angle for control rats showed a preference for the correct angle, whereas vestibular lesioned rats did not show this preference. This error in initial heading suggests impaired angular path integration, which is thought to depend on the HD signal (discussed in Section “HD Signal Involvement in Navigation – Electrophysiology and Behavior”). Although the homing task’s sole reliance on self-movement information has been questioned, a study confirmed that self-movement cues can indeed guide navigation on this type of homing task (van der Meer et al., 2010). In their experiment rats had to traverse a circular table to a central platform, after which the table or central platform was rotated in order to displace the original start location relative to the rat. After this rotation the rats had to return to their initial starting location. For most rotation trials, the return trip led to a location that was consistent with the rotation, instead of to the original location as defined by allocentric cues. This result confirms the use of self-movement cues for homing, and the fact that vestibular lesioned rats were impaired on homing tasks, provides strong evidence for a vestibular contribution to non-visual navigation. Further, the vestibular system appears to contribute to navigation performance on relatively simple navigation tasks. Bilateral sodium arsanilate injections to the vestibular labyrinth impaired rats’ ability to find a water cup that was consistently located in one corner of a square arena when visual landmarks were absent (Stackman and Herbert, 2002). When visual landmarks were available, however, vestibular lesions did not disrupt the ability to find the water cup. Thus, we currently have considerable evidence that the vestibular system provides some of the idiothetic cues that guide navigation when familiar visual landmarks are not available.

The results of navigation tests following complete vestibular lesions clearly indicate an important role for the vestibular system in both visual and non-visual navigation, but the specific type of vestibular contribution (rotational vs. linear movement) cannot be gained from these studies. Spatial deficits in animals with bilateral labyrinthectomy could have resulted from impaired sensation of gravity, translation, or rotation, given that signals from both the otolith organs and semicircular canals were eliminated in lesion studies. An early study indicated that the semicircular canals contribute to the ability of female gerbils to collect their pups and carry them directly back to the nest in complete darkness (Mittelstaedt and Mittelstaedt, 1980). When the dam was slowly rotated (acceleration ≤0.24°/s^2^) to an angular displacement of 37° after arriving at the pups’ location, the return trip deviated by a similar angle. However, rapid rotation did not impair the direct return to the nest, suggesting the semicircular canals detected the rapid rotation and allowed her to correct for the rotation. Additional evidence for a vestibular contribution to non-visual navigation comes from a study that used a six-arm radial maze where rats could be rotated between trials (Matthews et al., 1989). When the center platform was rotated in the absence of landmark cues, control rats were able to detect the passive rotation and choose the correct arm, whereas vestibular lesions impaired this ability. The addition of visual landmark cues improved performance somewhat for the vestibular group, but only partially compensated for the loss of vestibular signals. These findings thus suggest that signals from the semicircular canals contribute significantly to navigation, but they do not address the possible otolithic contribution.

Several recent studies have tested the otolithic contribution to navigation by using genetically modified mice that lack functional otolith organs. One study evaluated the open-field behavior in darkness of *headbanger* mice, which have a congenital malformation of the stereocilia in the utricle (Avni et al., 2009). Control animals typically traveled over long paths and visited many parts of the arena, whereas *headbanger* mice typically made many short movements, followed by a return to the start location. In contrast, control mice moved throughout the arena in relatively continuous journeys, thus visiting more zones than *headbanger* mice. This finding suggested that the *headbanger* mice had impaired path integration – the navigational process by which animals update their spatial orientation by continuously monitoring their movement cues (e.g., vestibular, proprioceptive, motor efference, optic flow). Interestingly, and somewhat surprisingly, a recent study showed that the otolith organs also contributed to navigation when it required the use of visual extramaze cues (Kirby and Yoder, 2012). Across days, homozygous *tilted* mice showed more spatial working and reference memory errors on a radial arm maze discrimination task than their heterozygous littermates when only extramaze cues were available, but performed as well as control mice when visible intramaze cues were available to signal the goal locations. Together, these two studies suggest the otolith organs contribute to spatial performance under both dark and light conditions. However, the fact that *tilted* mice were not impaired at cued navigation on a radial maze suggests they were able to recognize locations when local cues were available. One possibility is that these navigation deficits resulted from impaired place recognition, which was recently demonstrated in otoconia-deficient *head tilt* mice (Machado et al., 2012). The navigation deficits in animals with otolith dysfunction thus appear to be specific to tasks that require the use of distal or self-movement cues. However, whether these deficits resulted from the lack of otolith signals or from changes to the semicircular canal-mediated signals is not known at the present time, given that congenital otolith deficiency may alter canal-mediated signals (Beraneck and Lambert, 2009). The canals and otolith organs provide convergent input to many neurons in the medial vestibular nuclei (Bush et al., 1993), and the lack of otolith input could produce altered responses in these cells. If true, canal-dependent functions such as the VOR could also be altered. This possibility was confirmed by a study using *head tilt* mice, which have dysfunctional otolith organs (Harrod and Baker, 2003). Nevertheless, the use of mice with dysfunctional otolith organs in spatial tasks provides important insight that is not possible to obtain from other models.

## VESTIBULAR PATHWAYS TO SPATIAL FUNCTIONS

### HIPPOCAMPAL THETA RHYTHM

Several studies have shown that vestibular signals may influence navigation via their involvement with hippocampal theta rhythm. First, rats with sodium arsanilate-induced vestibular lesions performed as poorly on the radial arm maze task as rats with hippocampal lesions, but animals with combined vestibular/hippocampal lesions did not perform worse than either lesion alone, suggesting the vestibular contribution to spatial performance may arrive via hippocampal circuits (Allen et al., 2007). This suggestion is consistent with the finding that vestibular lesions attenuate hippocampal theta rhythm – a robust field potential oscillation associated with sensory and movement processing (Bland and Oddie, 2001; Russell et al., 2006). Second, passive rotation induced continuous theta rhythm in rats and mice (Gavrilov et al., 1995; Shin, 2010) via the activation of cholinergic septohippocampal cells (Tai et al., 2012). It is important to note, however, that the attenuation of movement-related theta rhythm alone, which can result from selective cholinergic or GABAergic lesions in the medial septum (Lee et al., 1994; Yoder and Pang, 2005), is not sufficient to impair spatial learning, as landmark navigation remained mostly unaffected on a water maze and radial arm maze following either of these lesions (Berger-Sweeney et al., 1994; Torres et al., 1994; Baxter et al., 1996; Pang et al., 2001; for review, see Parent and Baxter, 2004). Further, a recent study demonstrated that theta-paced stimulation alone could not restore vestibular lesion-induced impairments in a spatial non-matching-to-sample task (Neo et al., 2012). These results suggest that theta rhythm normally includes more than just vestibular information, although the vestibular content of theta rhythm is not well understood at the present time. However, it remains possible that vestibular information is conveyed by both theta rhythm and the HD signal, and both of these signals are necessary for normal spatial performance (discussed in Section “Spatial Signals”). Both the ascending theta and HD cell circuits originate in the tegmental region – which receives vestibular signals – and include many of the same nuclei, albeit different subregions (for review, see Vertes et al., 2004). Future studies focusing on the vestibular involvement in hippocampal physiology and spatial signals will undoubtedly provide important advances in our understanding of the sensory signals that contribute to spatial functions.

### SPATIAL SIGNALS

**FIGURE 1:**
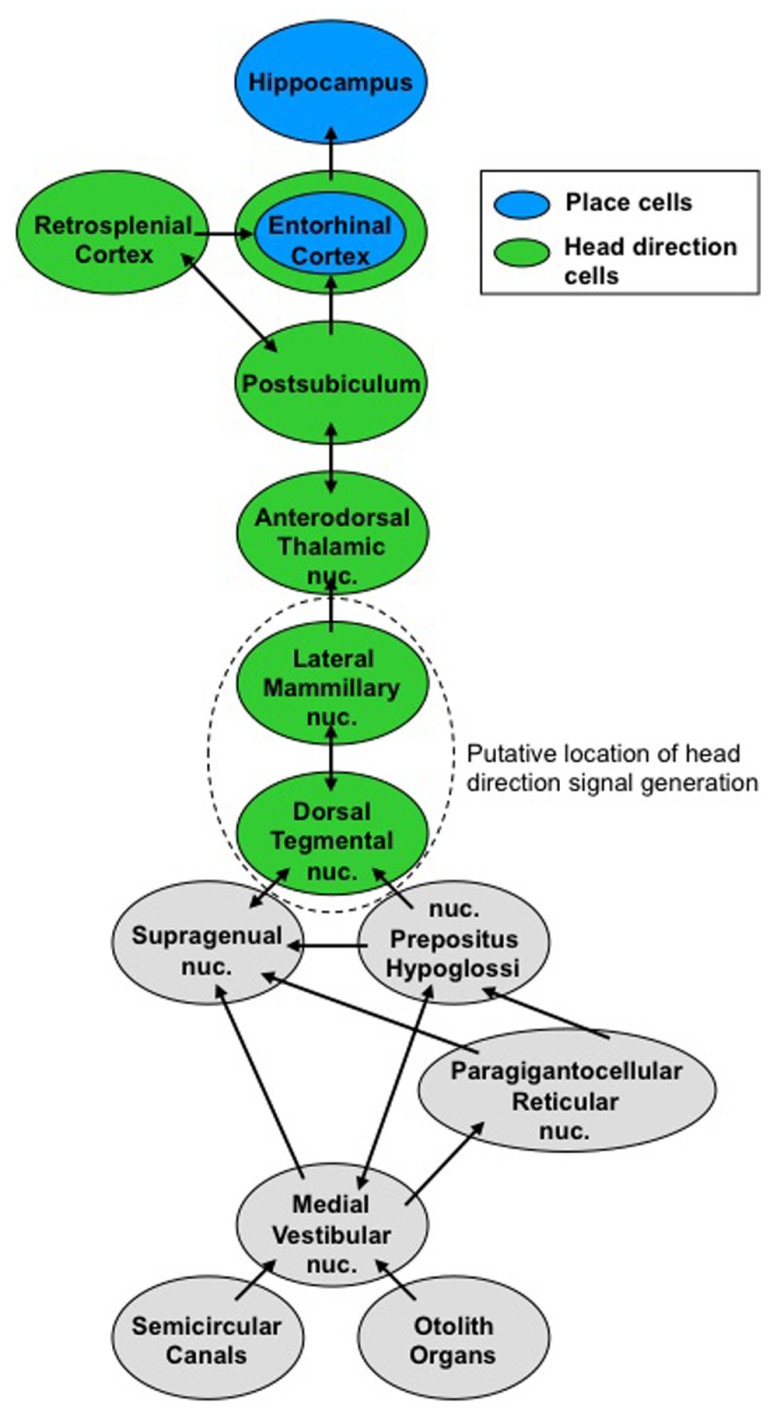
**Vestibular pathways to the ascending head direction cell circuit.** Signals from the semicircular canals and otolith organs converge on the medial vestibular nuclei. This vestibular information contributes to the head direction signal, which ascends to brain regions involved in navigation.

The vestibular system may contribute to spatial performance via its influence on generating various spatial signals. The pathway that conveys vestibular information to the hippocampus and place cells is not well known, but we have a much better understanding of the vestibular pathway to the HD cell circuit. Generation of the HD signal is thought to occur within the reciprocal connections between the dorsal tegmental nuclei (DTN) and lateral mammillary nuclei (LMN), based on the representation of angular head velocity, which the DTN receives from the vestibular nuclei via the supragenual nucleus and the nucleus prepositus (Lannou et al., 1984; Brown et al., 2005; Song and Wang, 2005; Biazoli et al., 2006). Once generated, the HD signal is projected bilaterally via the mammillothalamic tract to the anterior dorsal thalamus (ADN), which then projects to the postsubiculum (PoS; also referred to as dorsal presubiculum). In turn, the PoS projects to the entorhinal and retrosplenial cortical areas (**Figure 1**). This view is supported by studies that demonstrated complete loss of the HD signal in the ADN after lesion of the DTN or LMN (Blair et al., 1999; Bassett et al., 2007; Sharp and Koester, 2008), and loss of direction-specific firing in PoS cells after lesion of the ADN (Goodridge and Taube, 1997). Given the known efferent connections of the PoS (van Groen and Wyss, 1990b), the HD signal in entorhinal and retrosplenial cortical areas presumably depends on input from the PoS and/or the ADN, although no data are available to support this notion. Additionally, vestibular information may reach high-level brain regions via other pathways, such as the ascending vestibular projections to the ventral posterior thalamus, which then projects to the parietal insular vestibular cortex (PIVC; for review, see Shinder and Taube, 2010). This pathway shares information involving proprioceptive somatic cues and may be more involved with postural awareness – particularly in distinguishing active head movements from head movements that accompany body movements. To date, this pathway has not been identified in rodents, but unilateral or bilateral parietal cortex lesions disrupt spatial performance on some tasks (Crowne et al., 1992; Kolb et al., 1994; McDaniel et al., 1995), and parietal cortical neurons are known to represent route-based information that may contribute to spatial task performance (Nitz, 2006, 2009). Further, human studies have revealed an important interaction between the parietal cortex and hippocampus during allothetic cue processing (Zhang and Ekstrom, 2013). Nevertheless, if these pathways do contribute to spatial performance, they occur separately from the HD signal because lesions of the parietal cortex do not disrupt the HD signal within the limbic system (Calton et al., 2008). Thus, at least a portion of the vestibular contribution to navigation appears to arrive at high-level brain regions via the HD signal, whereas the other ascending vestibular pathways most likely contribute to other functions, such as postural awareness.

The importance of the vestibular system in contributing information to various types of spatial signals has been well demonstrated both directly and indirectly (**Table 1**). One of the first studies showed that the HD signal was disrupted by bilateral lesions of the vestibular labyrinth (Stackman and Taube, 1997). The authors were unable to identify any HD cells in the ADN in rats that had previously received bilateral injections of sodium arsanilate into the vestibular apparatus. An additional inactivation study confirmed these results and further demonstrated vestibular involvement in the activity of hippocampal place cells (Stackman et al., 2002). This study recorded HD cells in the PoS and place cells in hippocampus, after which tetrodotoxin was injected bilaterally into the vestibular apparatus. Post-injection recordings showed that this temporary vestibular inactivation disrupted the HD and place cell signals for several days, after which the signals recovered. The loss of location-specific activity in hippocampal place cells after removal of vestibular signals was confirmed in rats following permanent bilateral vestibular lesions (Russell et al., 2003b). Interestingly, the vestibular contribution to the place cell signal does not appear to be conveyed exclusively via the HD signal, as lesions of the ADN (Calton et al., 2003) or LMN (Sharp and Koester, 2008) impaired the stability of hippocampal place cells, but did not disrupt their location-specific activity. Nevertheless, these studies indicate an important role of the vestibular system in generating both place and HD cell signals.

Additional studies have indirectly confirmed that vestibular signals contribute to the HD signal. One study evaluated HD and place cell activity when self-movement cues and visual landmarks were in conflict (Knierim et al., 1998). Both HD and place cells were predominantly controlled by visual landmarks when the conflict was small, but self-movement cues provided the greatest influence when the conflict was large. In normal situations where self-movement and visual cues are not in conflict, discrete visual landmarks often predominantly control the HD signal (Goodridge and Taube, 1995). However, in the absence of discrete cues, the HD signal can be controlled by geometric information – which serves as a landmark – or by self-movement cues. When rats were permitted to actively explore asymmetric environments, the HD signal was controlled by self-movement cues instead of geometry (Knight et al., 2011). However, when rats were passively disoriented between trials, HD cells were predominantly controlled by geometric cues (see also Clark et al., 2012b). Given the vestibular involvement in path integration and the HD signal, at least a portion of the self-movement information that controls the HD signal likely originates in the vestibular system. Overall, the results of lesion studies, disorientation studies, and cue conflict studies demonstrate an important role for vestibular representations in both place cell and HD cell signals.

The loss of spatial signals after elimination of vestibular information suggests that spatial signals depend on information from the otolith organs or semicircular canals, or both. The first study to address which component of the vestibular system might be important was an experiment recording from hippocampal place cells on the Neurolab space shuttle mission, where the microgravity (0-*g*) environment causes reduced gravitational activation of the otolith organs (Knierim et al., 2000). The microgravity environment was found to have minimal effect on place cells as rats navigated a three-dimensional track. This finding suggests that otolith signals are not necessary for generating place cell activity, at least in terms of providing a tonic gravitational signal. The otolith organs, of course, would have detected translatory movements in the 0-*g* environment, and this representation of translation may therefore have provided a critical component of the place cell signal. However, a recent study suggests that place cell firing can persist in virtual navigation, despite the absence of translation and associated activation of the otolith organs (Chen et al., 2013). This finding suggests that the representation of translation is not necessary for place cell function, although the number of complex spike cells that fired in a location-specific manner was reduced in virtual navigation, relative to real-world navigation in a similar environment. Real-world translation may therefore contribute to the place cell signal, but no previous studies have shown whether this contribution depends specifically on the otolith organs. To address this issue, we recently recorded from hippocampal cells in otoconia-deficient *tilted* mice and found a small number of relatively normal place cells while the mice foraged in an open field, suggesting the otolith organs are not necessary for the place cell signal (Yoder et al., 2014 Midwestern Psychological Association). Aside from these studies, no evidence is currently available to disambiguate the otolith and canal contributions to the place cell signal.

Other experiments have addressed the otolithic contribution to the HD signal. One study evaluated the effects of microgravity on HD cell activity by recording from rats during parabolic flight (Taube et al., 2004). HD cells remained directional when rats navigated on the floor during 0-*g*, suggesting the otolithic representation of gravity is not crucial for the HD signal. However, if the animal was passively moved to the walls or ceiling of the apparatus the directional signal was lost, suggesting that the representation of gravity contributes to reorientation when the plane of locomotion changes. It is also possible that this HD signal instability occurs only during passive displacement in conditions where the lack of gravity detection prevents constant monitoring of body orientation. This issue was later addressed by a study in 1-*g* on Earth that monitored HD signal activity during active locomotion in upright, pitched, and inverted positions (Calton and Taube, 2005). Rats were trained to shuttle from one compartment of an apparatus located on the floor, up a wall, along the underside of the ceiling, and down the opposite wall into a second compartment of the apparatus, after which the animals reversed direction to shuttle back to the original start compartment. During this journey, many HD cells remained directional when the rat ascended the first wall but lost their directional tuning when the rat became inverted on the ceiling, only to regain directionality when the rat descended the second wall to reach the goal compartment on the floor. We have since confirmed that HD cells lose their directional firing during inversion using a different spatial task. In this task rats had to find an escape hole while walking inverted along the underneath side of a large suspended circular platform (Gibson et al., 2013). This loss of directional firing during inversion may have resulted from an unfamiliar otolith signal, given that inversion is rarely experienced by terrestrial lab rats. Importantly, along with the parabolic flight experiment, this result indicates that HD signal degradation occurs during inversion, regardless of whether the animal is actively or passively displaced and whether the animal is in a 0-*g* or 1-*g* environment. More recently, a complementary test of the otolithic contribution to the HD signal used mice lacking functional otolith organs (Yoder and Taube, 2009). HD cells in *tilted* mice were robustly directional during the initial recording session, but became progressively degraded across trials. Additionally, many non-directional “bursty” cells were present in *tilted* mice during recording sessions when no HD cells were found. This bursty pattern was similar to that of HD cells, which show bursts of activity when the head is pointed in a particular direction, although the activity of bursty cells failed to reliably occur while the head was pointed in only one direction. These bursty cells may therefore have been HD cells that failed to maintain precise directional tuning. Nonetheless, the HD signal degradation of *tilted* mice may have resulted from the absence of a subjective vertical, which appears to depend on the detection of gravity (Mittelstaedt, 1986). Thus, the loss of otolithic signals is detrimental to the HD signal, but does not account for the entire effect of vestibular lesions on the HD signal. It is therefore possible that the semicircular canals provide the information crucial to HD signal generation.

**FIGURE 2:**
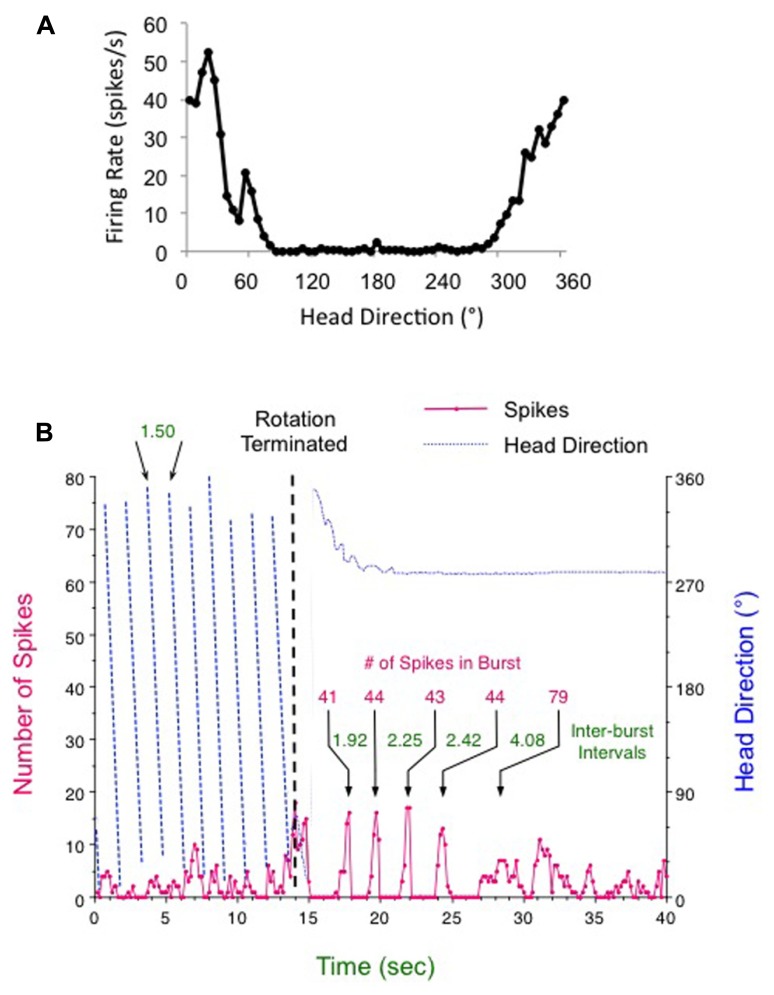
**Head direction cell responses following (A)** an active foraging session in the cylinder and **(B)** continuous clockwise rotation for 1 min (only the final 14 s are shown) followed by abrupt braking (vertical dashed line). After termination of rotation the HD cell fired in five periodic bursts over about 10 s. For this post-rotation period the amount of time in between each burst increased over each interval (times denoted in green for each inter-burst interval), although the number of spikes composing each burst (denoted in red) was about the same – particularly for the first four bursts. See text for further details.

Involvement of the semicircular canals in the HD signal is indicated by several recent studies. **Figure 2** presents data from our laboratory demonstrating that HD cells respond with periodic bursts of firing following post-rotational spinning of the rat (Taube, 2004 Barany Society Meeting). First, a typical HD cell in the ADN was recorded while the rat foraged for food within a cylinder containing a prominent visual cue card (**Figure 2A**). Then the rat, which was free to move on a 1-m diameter platform, was spun continuously in the dark in one direction for about 1 min at constant speed (~30–50 rpm). The rat generally remained motionless during the rotational period and did not move or turn its head. After a few seconds of rotation, there was a loss of direction-specific firing in the HD cell, although the cell was not quiescent, but appeared to fire at random directions leading to an elevated background firing rate (see **Figure 2B**, pre-brake period red line). The loss of directional tuning was presumably due to the disoriented state of the animal as a result of the continuous rotations. The platform was then stopped abruptly (**Figure 2B**, vertical dashed line) while cell recording continued during the post-rotational period. During the post-rotational period the rat initially moved for the first 3 s (14–17 s time points, see blue dotted line in **Figure 2B**) and then remained motionless over the next 23 s. The red line in **Figure 2B** shows that the cell’s response during the rotational period (from 0 to 14 s) occurred at a relatively steady low firing rate that was generally non-directional. The firing rate during this period was greater than the background firing rate (0.378 spikes/s) of the cell during an active foraging session without any spinning (**Figure 2A**). Once the rotation was stopped at the 14 s time point, the cell responded by going through an initial burst of firing (at the 14–15 s time point), which was followed by five periods of burst firing (between the 15–25 s time points). Interestingly, each burst contained about the same number of spikes (~44) and lasted about 666 ms. The period in between each burst was marked by the absence of firing and the interburst interval increased in a linear manner (*r* = 0.992) over 10 s. Following the 13 s post-rotational period, the cell’s firing rate became more variable and was somewhat increased compared to the period during the rotational spinning. After ~1 min, the cell’s firing pattern returned to normal and was once again directional (data not shown). These data suggest that the HD signal can be heavily influenced by canal activation, which would have occurred when the canal’s cupula was deflected by the inertial movement of the endolymph fluid after the head rotation had stopped abruptly. The cupula’s deflection would have activated vestibular hair cells and led to a response that was propagated forward and affected the HD cell network to the extent that the animal perceived it was spinning (in the opposite direction) and the recorded HD cell was activated periodically – once for each revolution through the cell’s preferred firing direction. The increasing interval between each burst was most likely due to the slowing of the endolymph fluid over time. Similar responses are seen with ocular nystagmus following termination of continual rotation (Leigh and Zee, 2006). Although this response did not occur in every animal every time the animal was rotated, the fact that it can occur demonstrates the importance of the vestibular system in generating the HD cell signal.

Other studies in animals with dysfunctional canals have confirmed the canals’ role in HD signal generation. One study used chinchillas, which have large vestibular apparatus that can be accessed easily during surgery, to determine whether removal of the canals would affect the HD signal (Muir et al., 2009). HD cells similar to those of rats were recorded from the chinchilla ADN prior to a surgery in which the horizontal, superior + posterior, or all three semicircular canals were plugged bilaterally, rendering the respective canals dysfunctional. After surgery, cells recorded within the ADN were no longer directionally tuned, suggesting the canals provide input that is crucial to the HD signal. Findings consistent with these results were provided by a study using *epistatic circler* (*Ecl*) mice, which have a genetic mutation that causes them to develop without horizontal canals – control mice exhibited normal HD cells, but no HD cells were detected in the ADN of *Ecl* mice (Taube and Valerio, 2012). Importantly, in both canal-plugged chinchillas and *Ecl* mice, many animals showed bursty cells similar to those of *tilted* mice. These would-be HD cells therefore appear to be able to maintain their basic firing properties, but lack the input necessary to maintain their directional specificity over time. This interpretation is quite plausible, given that the canals represent changes in angular head position along the horizontal plane, which would move the head from one direction to another. This direct coupling between the canals and the generative portion of the HD circuit may therefore be akin to the coupling between the canals and VOR circuit and presumably involves a mathematical integration in time to derive angular head displacement (i.e., a change in HD) from the angular head velocity signal that is ubiquitous throughout vestibular brainstem pathways. Whether the nucleus prepositus, which is critically involved in the mathematical integration for the VOR, is also involved in the mathematical integration for the HD system is not known. Preliminary studies, however, have shown that lesions of the nucleus prepositus disrupt the HD signal (Butler and Taube, 2012). Other possibilities include the supragenual nucleus, which lies anatomically adjacent to the medial vestibular nuclei, and is also known to be a critical structure for generating the HD signal (Clark et al., 2012). Finally, a preliminary study reported that velocity storage, the prolonged vestibular signal within the vestibular nuclei that outlasts the afferent sensory signal, contributes to stable HD representations. Taube and Bassett (2005) found that disrupting velocity storage, by severing the commissural connections between the two vestibular nuclei, resulted in a constant under-signaling of angular head velocity at high frequency head turns. In turn, this effect led to a distorted HD signal where the animal’s perceived directional heading consistently lagged behind its true (actual) heading. This occurrence was manifested by the HD cell shifting its preferred direction ahead as the animal moved through the environment.

Overall, the vestibular system is known to contribute to theta rhythm, place cells, and HD cells, but it is also possible that theta rhythm directly influences these spatial representations. The relation between theta rhythm and place cell activity is well known, with place cells showing “phase precession,” or a tendency to fire at increasingly earlier phases of the theta cycle as the animal walks through a cell’s place field (O’Keefe and Recce, 1993). In contrast, HD cells in the ADN, as well as the LMN and PoS, do not show theta phase precession, and an interspike interval analysis of these cells did not reveal theta rhythmic modulation (Taube, 2010). However, some HD cells in the anteroventral thalamus (AVN) did show theta modulation, suggesting a relation between theta rhythm and HD cells in some brain areas (Tsanov et al., 2011). This theta modulation may arrive at AVN via a direct projection from the medial mammillary nuclei, where many angular head velocity cells are strongly modulated by theta rhythm (Sharp and Turner-Williams, 2005). Theta modulation of HD cell activity has also been observed in the entorhinal cortex, with some HD cells firing preferentially on alternate theta waves (i.e., “theta skipping”), while other HD cells within the same region fail to show this firing pattern (Brandon et al., 2013), suggesting that there are different types of HD cells within the entorhinal cortex. Theta skipping is known to occur throughout the entorhinal cortex (Deshmukh et al., 2010), but the significance of this phenomenon is not known at the present time. Thus, theta rhythm is heavily involved in place cell function, but its influence on the HD signal is less pronounced and is not found throughout the HD cell circuit.

## HD SIGNAL INVOLVEMENT IN NAVIGATION – LESION STUDIES

The vestibular system is necessary for both the HD signal and navigation, suggesting that the HD signal may convey vestibular information to higher cortical navigation centers. If true, disruption of the HD signal at any point along the ascending HD circuit (DTN → LMN → ADN → PoS) would impair navigational performance. This proposal has been confirmed by lesion and inactivation studies using various spatial tasks.

### DORSAL TEGMENTAL NUCLEI

*N*-methyl-D-aspartate (NMDA) lesions of the DTN impaired both landmark navigation and path integration on a food-carrying task where sighted or blindfolded rats walked from a refuge into an open field, foraged for large food pellets in the open field, and then carried the food back to the refuge for consumption (Frohardt et al., 2006). This study evaluated task performance after DTN or ADN lesions, and found that the greatest impairment was seen in the DTN lesioned animals, both with and without blindfolds. DTN involvement in spatial functions was recently confirmed with DTN lesion-induced impairments on a water T-maze task that required rats to choose the direction that led to an escape platform, as well as on a food-foraging task in light (Dwyer et al., 2013). Additionally, in a recent study that manipulated various local and distal cues in the water maze task, DTN lesions increased the number of trials it took the rats to reach criterion and impaired their ability to navigate accurately to a place in absolute space, relative to a set of stable distal landmarks (Clark et al., 2013). Interestingly, the DTN lesioned animals were not impaired on the water task when the pool was repositioned at the start of a trial, but the platform was placed in the same relative position within the pool. Thus, despite the absence of an HD signal, the rats were still capable of swimming in the proper direction relative to cues located within the pool.

### LATERAL MAMMILLARY NUCLEI

The other region of the HD generator, the lateral mammillary nuclei, is also necessary for navigation. Mammillary lesions impaired reinforced T-maze alternation and impaired the use of spatial cues in a cross-maze and a radial-arm maze, but did not impair performance on an egocentric discrimination task (Neave et al., 1997). Another study showed that neurotoxic mammillary, as well as anterior thalamic and fornix lesions, disrupted forced and continuous T-maze alternation (Aggleton et al., 1995). A series of studies confirmed these results, with mammillary (and anterior thalamic) lesions impairing performance on a radial arm maze, but not an egocentric conditional associative learning task (Saravis et al., 1990; Sziklas and Petrides, 1993, 2000, 2004; Sziklas et al., 1996; for review, see Sziklas and Petrides, 1998). LMN lesions also moderately impaired performance on a water maze task, although the deficits tended to be transient and were not as great as when the entire mammillary nuclei, including the medial mammillary nuclei, were lesioned (Vann, 2005). The fiber tract that conveys the mammillary signal to the thalamus is also critical for spatial performance, as lesion of the mammillothalamic tract or mammillary bodies impaired forced alternation on a T-maze, and impaired radial arm maze and water maze performance (Vann and Aggleton, 2003). A more recent study demonstrated that mammillothalamic tract lesions disrupt performance on a food-hoarding task that requires path integration and the processing of self-movement (idiothetic) cues for accurate performance (Winter et al., 2011).

### ANTERODORSAL THALAMUS

As one would predict, the ADN, which receives the HD signal from LMN (Blair et al., 1999; Bassett et al., 2007), is also involved in navigation. NMDA lesions of the entire anterior thalamic region impaired radial arm maze performance as well as allocentric cue-based alternation and forced alternation on a T-maze, but did not impair egocentric alternation (Aggleton et al., 1996). This study also evaluated performance as a function of the lesion extent, with groups having damage limited to the anteromedial nuclei or the anterodorsal/anteroventral nuclei. Neither group showed impaired allocentric alternation on the cross-maze, but the anteroventral/anterodorsal group was impaired on the radial maze task. Anterior thalamic lesions also impaired place learning, but not visual discrimination, on a water maze task (Moreau et al., 2013).

In finding the hidden platform in a water maze task, an animal can rely on the absolute reference frame of the room as indicated by distal room cues – mostly visual landmarks along the walls of the surrounding room, or on the relative reference frame, which is based more on the local, intramaze cues, such as the distance between the apparatus wall and the platform. A recent study in mice reported that the ADN played an important role in processing local cue information (Stackman et al., 2012). Examination of the swim paths of male control mice indicated that the animals relied on pool-based cues (relative reference frame) over extramaze cues (absolute reference frame) on a probe test that did not contain the platform. However, rotation of the extramaze cues led to a corresponding shift in the trajectories the mice took when released into the pool, suggesting that both pool- and room-based reference frames were used to guide behavior in the search for the hidden platform. Disorienting the mice before placement in the water disrupted their reliance on pool-based cues and responding to a relative reference frame. Mice that had their anterior thalamic nuclei inactivated showed a preference for using an absolute reference frame strategy. In contrast, dorsal CA1 region inactivation did not disrupt the preference for the relative reference frame (intramaze cues). Taken together, these results suggest that the anterior thalamic nuclei play an important role in guiding behavior based on the relative, local cue-based reference frame, as opposed to a strategy based on the absolute, distal-cue based reference frame. Because the PoS has been suggested to be the route by which landmark information is conveyed to the hippocampus (Goodridge and Taube, 1997; Yoder et al., 2011b), it is possible that inactivation of the anterior thalamic nuclei would not interfere with the information transfer of distal landmark information to the hippocampus – thus, leaving intact the absolute reference frame-based strategy that was evident in the experimental group.

### POSTSUBICULUM

Additional evidence for the HD signal’s role in navigation is provided by studies of navigation in animals with PoS lesions. Both NMDA and electrolytic PoS lesions impaired radial arm and water maze performance, but did not impair performance on a non-spatial cued version of the water maze or on a conditioned food aversion task (Taube et al., 1992). PoS lesions also impaired spatial alternation on a T-maze when the second trial was delayed (Bett et al., 2012). However, this study also showed that PoS lesions did not impair homing on a food-hoarding task (but see Valerio et al., 2011 for an opposite account). The intact homing after PoS lesions is particularly interesting, given the Frohardt et al. (2006) report (mentioned above) of impaired homing after DTN or ADN lesions. Together, these results suggest that HD signal generation is necessary for homing, but this function does not depend on the transfer of HD information out of PoS. Another possibility is that the direct projection from ADN to retrosplenial cortex (van Groen and Wyss, 1990a, 2003), which may include the HD signal, is sufficient to support path integration, given that the retrosplenial cortex contains HD cells (Chen et al., 1994; Cho and Sharp, 2001) and is necessary for path integration (Cooper and Mizumori, 1999; Whishaw et al., 2001).

In addition to its role in navigation, the PoS also contributes to functions other than the HD signal. A recent study found that blockade of PoS kainate and AMPA receptors with CNQX disrupted the stability of hippocampal place cells, whereas AMPA/kainate receptor blockade or NMDA receptor blockade with D-AP5 impairs object-location memory (Bett et al., 2013). PoS lesions also impaired the acquisition of contextual fear, and impaired both the acquisition and expression of auditory conditioned fear (Robinson and Bucci, 2012). Thus, it remains unclear whether the predominant PoS contribution to navigation involves the transfer of the HD signal to cortical structures or the transfer of object-location information to the HD and other spatial signals. We can conclude, however, that the HD signal is necessary for navigation, as HD signal disruption at any point within the ascending HD circuit disrupts navigation.

## HD SIGNAL INVOLVEMENT IN NAVIGATION – ELECTROPHYSIOLOGY AND BEHAVIOR

The importance of the HD signal for navigation is indicated by lesion studies, but its exact contribution to navigation is not well understood at the present time. One possibility is that the HD signal serves as a reference (or compass) by which animals navigate. If true, the HD signal would remain constant within a given environment and navigation would change relative to the HD signal (and thus, the environment) based on reward contingencies. A study of HD cell activity during navigation on parallel T-mazes supports this idea (Dudchenko and Zinyuk, 2005). In T-mazes located in adjacent rooms, an HD cell’s preferred firing direction remained constant when the mazes were parallel, but reoriented to the second maze when it was rotated 90° relative to the first maze, suggesting that the HD signal was controlled by the maze instead of by distal room cues. A more recent study found that both HD and grid cells in the entorhinal cortex were controlled by distal cues to a greater extent than by cues within the T-maze if the maze was navigated recently, but to a lesser extent if the maze was navigated at an earlier time (Gupta et al., 2014). Dudchenko and Zinyuk (2005) also provided evidence for HD signal involvement in path integration-based navigation. In an apparatus consisting of four interconnected chambers, the preferred firing direction remapped if the animal was passively transported between arenas, but remained stable if the animal walked between arenas. Similar results were found with the dual-chamber apparatus and a two-room maze, where passive transport from familiar to novel arenas caused HD signal remapping, but active locomotion maintained HD signal stability between arenas (Taube and Burton, 1995; Stackman et al., 2003; Yoder et al., 2011a). However, this compass-like representation across arenas does accumulate error over time and distance traveled in path integration tasks. For example, instead of remaining constant, the HD signal was updated relative to the environment when a previous journey contained errors (Valerio and Taube, 2012). Blindfolded rats were trained to perform a food-carrying task where the animal foraged for food and then relied on path integration during the outbound journey in order to calculate a direct return path back to the start box in order to consume the food. After training, rats were implanted with electrodes aimed at HD cells in the ADN. As predicted, there was a correlation between the amount of error in the HD signal (comparing the HD cell’s preferred direction in the refuge to its value at the onset of the return trip) and the amount of error in the rat’s behavioral trajectory. When the rat made an error in its return path, the cell’s preferred direction was usually “reset” to its initial value in the refuge. However, on some occasions – particularly when large errors occurred, the HD signal “remapped,” with the cell’s new preferred firing direction corresponding to the angular error experienced on the rat’s return path to the refuge. In other words, rather than correcting the error upon return to the refuge and “resetting” its preferred direction based on the known reference cues within the refuge, the cell adopted a new preferred direction based on how it fired during the rat’s erroneous return path to the refuge. This finding suggests that, although the HD can be controlled by path integration, the reward system can override this control in order to update the signal when necessary. This study therefore provides important insight into the relation between the HD signal and behavior, although it is presently unknown whether similar updating occurs during landmark navigation, which usually dominates the control of spatial signals. Future studies are therefore important to further elucidate the role of the HD signal in navigation, as well as the nature of the information it carries.

The vestibular system also appears to play an important role in the formation of a cognitive map. One study showed that when rats were inverted (upside-down), they had difficulty finding an escape hole from four different entry points, but can learn to solve the task when released from one or two entry points (Valerio et al., 2010). This spatial impairment could have resulted from the disrupted HD signal that occurs when rats navigate in an inverted position, as discussed above. To test this, a subsequent study monitored HD cell activity in the two entry-point version of the inverted task and when the rats were released from a novel start point (Gibson et al., 2013). The authors found that, despite the absence of direction-specific firing in HD cells, rats could successfully navigate to the escape hole when released from one of two familiar locations by using a habit-associated directional strategy. Moreover, in the continued absence of normal HD cell activity, inverted rats failed to find the escape hole when they started from a novel release point. These results suggest that the HD signal is critical for accurate navigation in situations that require a flexible representation of space, such as when using a cognitive mapping strategy, but not in situations that utilize habit-like associative spatial learning. Further, it was the direct result of being inverted and possibly experiencing an unfamiliar otolith signal that led to the disruption of direction-specific firing in HD cells.

## CONCLUSION

Overall, we currently have considerable evidence that the vestibular system contributes to spatial signals and navigation. The semicircular canals are necessary for HD signal generation, whereas the otolith organs support HD signal stability. Given the vestibular involvement in HD signal generation, the HD signal appears to convey at least a portion of the vestibular contribution to spatial functions. Our understanding of this contribution continues to develop as new approaches become available for the investigation of the roles of the semicircular canals and otolith organs in spatial cognition.

## Conflict of Interest Statement

The authors declare that the research was conducted in the absence of any commercial or financial relationships that could be construed as a potential conflict of interest.
